# Intraoperative tachycardia, hypercapnia, hyperthermia and muscle stiffness in a dantrolene unavailability case. Can calcium channel blockers be valuable?

**DOI:** 10.1186/1471-2253-14-S1-A8

**Published:** 2014-08-18

**Authors:** Acílio Marques, Catarina Dourado, Carla Retroz Marques, Raquel Cabral

**Affiliations:** 1 Department of Anaesthesiology, Coimbra University Hospital Centre, Coimbra, 3000-075, Portugal

## Background

The case reported involved the constellation of the above signs and exceptional circumstances meant that the right drug was not available at the time. Calcium channel blockers were given for life-saving reasons. However their value for that purpose is questionable and may be dangerous.

## Case report

Caucasian healthy man, 19 years old, was anesthetised with propofol bolus, remifentanil perfusion and sevoflurane maintenance. No muscle relaxants were used.

Tachycardia was treated at 30 minutes with propranolol EV. Hypercapnia was evident at 50 minutes. Hyperthermia was confirmed at 60 minutes and specific attitudes were taken: sevoflurane was stopped and replaced by propofol perfusion, O_2_ at 18 L/min, the patient was cooled, diuresis promoted and dantrolene was requested. At 70 minutes, muscle stiffness with ventilatory difficulties (P_aw_>40 cmH_2_O, CO_2ET_>65 mmHg, SpO_2_=82%), axillar temperature=40.5 ºC (0,2^o^C/min) - verapamil 5mg EV was given.

The arterial blood gases: pH=7.23, K^+^ =6.2mEq/L. Insulin and dextrose was used.

The dantrolene was delivered at 80min but the clinical improvement delayed their administration to 120min: 40 mg guided by K^+^ blood analysis. At 48h the patient had severe legs oedema and at 72h blood CK =12,700 U/L. He was discharged at one week and had fully recovered from renal and pereoneal nerve dysfunctions in the following months.

This patient was treated in the maxillofacial department, which is away from the main hospital unit. The dantrolene unavailability is an issue of the anaesthesiologist’s responsibilities. We did not carry out that specific checklist and were not aware of that situation. Portugal does not have laboratories for definitive diagnosis of malignant hyperthermia (MH) but the Larach clinical grading scale to predict patients susceptibility give 70 points; in the range of 50 points or more classified as almost certain. However, discussion of clinical signs and its sequential identification might question the therapeutic timing. If the therapy was implemented by the hypercapnia diagnosis, might the clinical evolution be different? And would the drug arrive in time for a life-saving situation? Is there any clinical indication for rechecking the local pharmacy?

We do not exclude the possibility that clinical improvement might have been a consequence of previous therapeutic measures; however, there was a temporal drug-effect.

The idea of using verapamil comes from experimental laboratory research showing how extracellular calcium affects the severity of MH and the pharmacologic applicability. That decision was taken from the life-saving perspective with available drugs knowing that a clinical-experimental shortcut was followed with inherent danger.

Probably the clinical use of this drug, for such purpose, is not repeatable, but its value in saving the patient’s life is obvious.

## Conclusions

Research and patient care partnerships are an ongoing process. There are various reasons why not all laboratory research translates into clinical applicability. However, mutual knowledge/information can be critical to solving issues relevant to a patient’s life. The value of calcium channel blockers when dantrolene was not available for this patient with suspected MH is one such successfully solved question.

